# Core hyphosphere microbiota of *Fusarium oxysporum* f. sp. *niveum*

**DOI:** 10.1186/s40793-024-00558-5

**Published:** 2024-03-09

**Authors:** Vanessa E. Thomas, Sanjay Antony-Babu

**Affiliations:** Department of Plant Pathology and Microbiology, Texas A&M University, College Station, TX 77843 USA

**Keywords:** Interkingdom interactions, Bacterial–fungal interactions, Pathobiome, Fusarium wilt

## Abstract

**Background:**

Bacteria and fungi are dynamically interconnected, leading to beneficial or antagonistic relationships with plants. Within this interkingdom interaction, the microbial community directly associated with the pathogen make up the pathobiome. While the overall soil bacterial community associated with Fusarium wilt diseases has been widely examined, the specific bacterial populations that directly interact with the Fusarium wilt pathogens are yet to be discovered. In this study, we define the bacterial community associated with the hyphae of Fusarium *oxysporum* f. sp. *niveum* race 2 (FON2). Using the 16S rRNA gene metabarcoding, we describe the hyphosphere pathobiome of three isolates of FON2.

**Results:**

Our results show a core microbiome that is shared among the three tested hyphospheres. The core hyphosphere community was made up of 15 OTUs (Operational Taxonomic Units) that were associated with all three FON2 isolates. This core consisted of bacterial members of the families, *Oxalobacteraceae*, *Propionibacteriaceae*, *Burkholderiaceae*, *Micrococcaceae*, *Bacillaceae*, *Comamonadaceae*, *Pseudomonadaceae* and unclassified bacteria. The hyphosphere of FON2 was dominated by order Burkholderiales. While all three isolate hyphospheres were dominated by these taxa, the specific OTU differed. We also note that while the dominant OTU of one hyphosphere might not be the largest OTU for other hyphospheres, they were still present across all the three isolate hyphospheres. Additionally, in the correlation and co-occurrence analysis the most abundant OTU was negatively correlated with most of the other OTU populations within the hyphosphere.

**Conclusions:**

The study indicates a core microbiota associated with FON2. These results provide insights into the microbe-microbe dynamic of the pathogen's success and its ability to recruit a core pathobiome. Our research promotes the concept of pathogens not being lone invaders but recruits from the established host microbiome to form a pathobiome.

**Supplementary Information:**

The online version contains supplementary material available at 10.1186/s40793-024-00558-5.

## Background

The hyphosphere, the zone of influence that immediately surrounds fungal hyphae, represents a major hotspot of interkingdom interactions between fungi and bacteria. Within the soil ecosystem, the hyphosphere functions as a major micro niche, where the exometabolic chemistry is influenced by both the host fungus and its hyphal-associated bacteria [1–3]. These molecular exchanges include genetic information, metabolic exchange and chemotaxis [3]. The bacterial physical association within the hyphosphere can be variable, including: (i) freely motile and moving along the hyphae attached as single cells, (ii) within the hyphae as endohyphal bacteria, or (iii) forming biofilms along the hyphae [3, 4]. The planktonic bacterial movement on the hyphae, termed the “hyphal highway” or “fungal highway” [5, 6], has been observed in several soil fungi. For example, the hyphosphere of *Lyophyllum* sp., a soil fungi, allows several individual bacteria to move along the hyphae and form biofilms [7]. The most well-studied bacterial–fungal interactions (BFI) system is between ectomycorrhizal fungi and its “helper bacteria” [8–11]. These systems demonstrate the triangular interaction of bacteria, fungi, and plants. This BFI can benefit the plant since fungal-associated bacteria can fix nitrogen and mobilize phosphates to enhance plant growth [10, 12]. While it is well established that beneficial fungal-associated bacteria can assist in plant nutrition, it is unclear whether such effects of bacteria are associated with plant pathogenic fungi. As a first step towards understanding the effect of a pathogenic fungi on the host-associated microbiome, we aim to define the microbial community structure of a soil-borne pathogen.

Previous research on the hyphosphere of pathogenic fungi focused on pathogen antagonism, with the goal of designing biocontrol measures [13]. However, knowledge of the hyphosphere bacteria associated with pathogen success is lacking. Evidences of bacterial influence on the virulence of pathogenic fungi [14] leads to a reasonable hypothesize that such effects are likely more pronounced in hyphosphere interactions, where the bacterial partner can help the host-fungi to express or enhance their virulence. Evidence of such interactions is known from endohyphal bacteria. For example, a *Burkhholderia* endosymbiont of rice seedling blight (*Rhizopus microspores*) produces phytotoxins that allow the fungal pathogen to infect the plant host [15] and an *Enterobacter *sp*.* Enhances the virulence of *Rhizoctonia solani* [16]. While these studies point to fungal-helper bacteria within the realm of pathogenesis, they are restricted to single bacterial taxa associated with one pathogen, and not consortia of microorganisms, as expected in a pathobiome.

The microbiome associated with the pathogen has been discussed as part of the biotic component of a relatively newer concept of pathobiome [17]. Although the functions of the microbial community within the pathobiome are unknown [18], we suggest the microbiota recruited in the pathobiome could potentially assist in infecting the hosts [16]. Thus, we address the recent suggestions to expand the traditional view of the plant disease triangle to the disease tetrahedral, to include a susceptible host biotic factor/microbiota (or symbiome) [19, 20]. We further add evidence to the ongoing paradigm shift from studying pathogens as lone invaders to recognizing disease at a holobiome level [17, 19, 21, 22].

In tis study, we set-out to investigate the hyphosphere bacterial diversity of the Fusarium wilt pathogen of watermelon. Fusarium wilts affect a wide range of crops and are caused by fungi classified in the *Fusarium oxysporum* species complex (FOSC). The FOSC strains are one of the most devastating plant pathogens, ranking fifth among the top ten most destructive plant pathogenic fungi [23]. The FOSC are further grouped into *formae speciales* (ff. spp.) based on host specificity [24, 25]. Within *formae speciales*, races may be designated, which differ in pathogenicity to cultivars or varieties of the host [26]. FOSC strains that are specific to watermelon are called *F. oxysporum* f. sp. *niveum* (FON). FON is widespread in areas with watermelon cultivation, including US states such as Florida, Georgia, and Texas, where farmers can experience 30–100% yield loss to this soil-borne pathogen [27, 28]. Race 2 of FON is widespread in these states [29, 30]. As a soil-borne pathogen, *F. oxysporum* f. sp. *niveum* race 2 (FON2) could interact with a diverse bacterial community, with the possibility of being neutral, antagonistic, or assisting in its virulence. As an initial step towards evaluating the hyphosphere bacteria acting as a pathobiome of FON, we investigated the diversity of the hyphosphere bacterial community of the fungus within infected plant tissue. We hypothesized that the bacterial associates of FON’s successful colonization of the host would best represent the pathobiome. We specifically asked the following questions: (1) does FON sustain a hyphosphere bacterial community?; (2) if so, can the fungus maintain consistent bacterial diversity through subsequent generations of in vitro subcultures?; (3) do various FON isolates have a central core bacterial community? In this study, we describe the hyphosphere bacterial assemblage of watermelon wilt Fusaria, using three different FON isolates from two counties in Texas, isolated between 2002 and 2020 (COM 2020, W2 2018, and HAR 2002). This study sets the stage to study hyphosphere bacteria of soil-borne fungi as potential pathobiome of the fungal pathogen.

## Methods

### Seed and soil sterilization

Watermelon seeds (hybrid ‘Super Gold’, Willhite Seed, Poolville, TX) were surface-disinfested as follows: Tween 20® (0.002%), 10% bleach, 70% ethanol and then rinsed with sterile water. Each operation lasted 2 min. The seeds were then placed on sterile 2% Gelrite (RPI) in Petri dishes. Aliquots of 100 μl of ¼ strength Hoagland’s solution were added to each seed [31]. Seeds were incubated in a dark box for up to 7 days at ambient room temperature (20°C-23°C). A soil mixture composed of 1:1—commercial sand; Jolly Gardener Pro-Line C/25 Growing Mix, in pots (9.8cm × 9.8cm × 8cm) was autoclaved (60 min fast exhaust) daily over three consecutive days. The soils were then moistened with 5 ml of sterile ¼X Hoagland’s solution into which the germinated seeds were planted. Plants were kept in a growth chamber with 13-h daily light.

### FON2 inoculum preparation

**Fig. 1:**
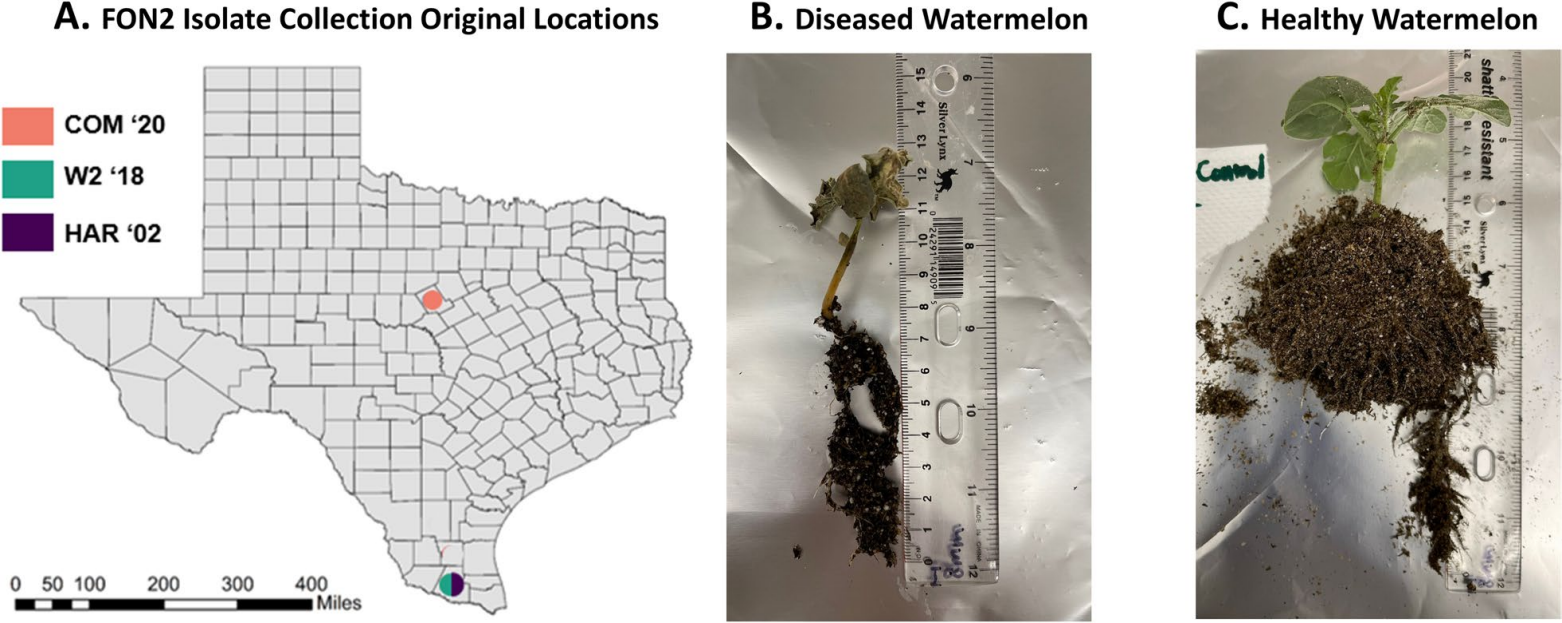
Isolates of FON2 (COM, W2 and HAR) were collected from different temporal and spatial backgrounds as shown in county map of Texas (**A**) with the locations and year of initial sampling. Wilting and necrotic symptoms of ‘Super Gold’ watermelon 10 days after inoculation with Fusarium oxysporum f. sp. niveum COM isolate (**B**) and healthy control (**C**)

We used 3 genetically-different FON2 isolates (COM, HAR, and W2) from our earlier study [32]. Briefly, COM was isolated in 2020, W2 in 2018, and HAR in 2002 (Fig. 1A). All Isolates were obtained from stems of symptomatic watermelon plants. The FON2 inoculum was prepared by culturing the strains in tryptic soy broth (TSB) supplemented with 100 mg/liter of streptomycin in flasks on an orbital shaker for 7 days at room temperature [32]. These isolates had no amplifiable bacterial DNA, using 27F-1492R primers [33], and, using ITS1-4 primers [34]their identities were confirmed as *Fusarium oxysporum* through nBLAST using the NCBI database [32]*.*

### Sand-oatmeal matrix inoculum

As a single experiment, as 10-g growth matrix consisting of 1:1 (w/w) oatmeal (The Quaker Oat Bran™): was dispensed into glass Petri plates and autoclaved for 20 min using the gravity cycle 121 °C. Following this, 200 μl of FON2 inoculum and 5 ml of 1 × phosphate buffer solution (PBS) were dispensed into the sand-oatmeal matrix and cultures were incubated at 27 °C for up to 7 days. Watermelon plants (6 per treatment) were inoculated after 5 days of growth after germination. To inoculate the plants, soil near the roots was removed without disturbing them. This soil was mixed with the sand-oatmeal fungal mix and placed back on the initial site of removal.

### Tissue sampling, and generation culturing from the hyphal tip

**Fig. 2:**
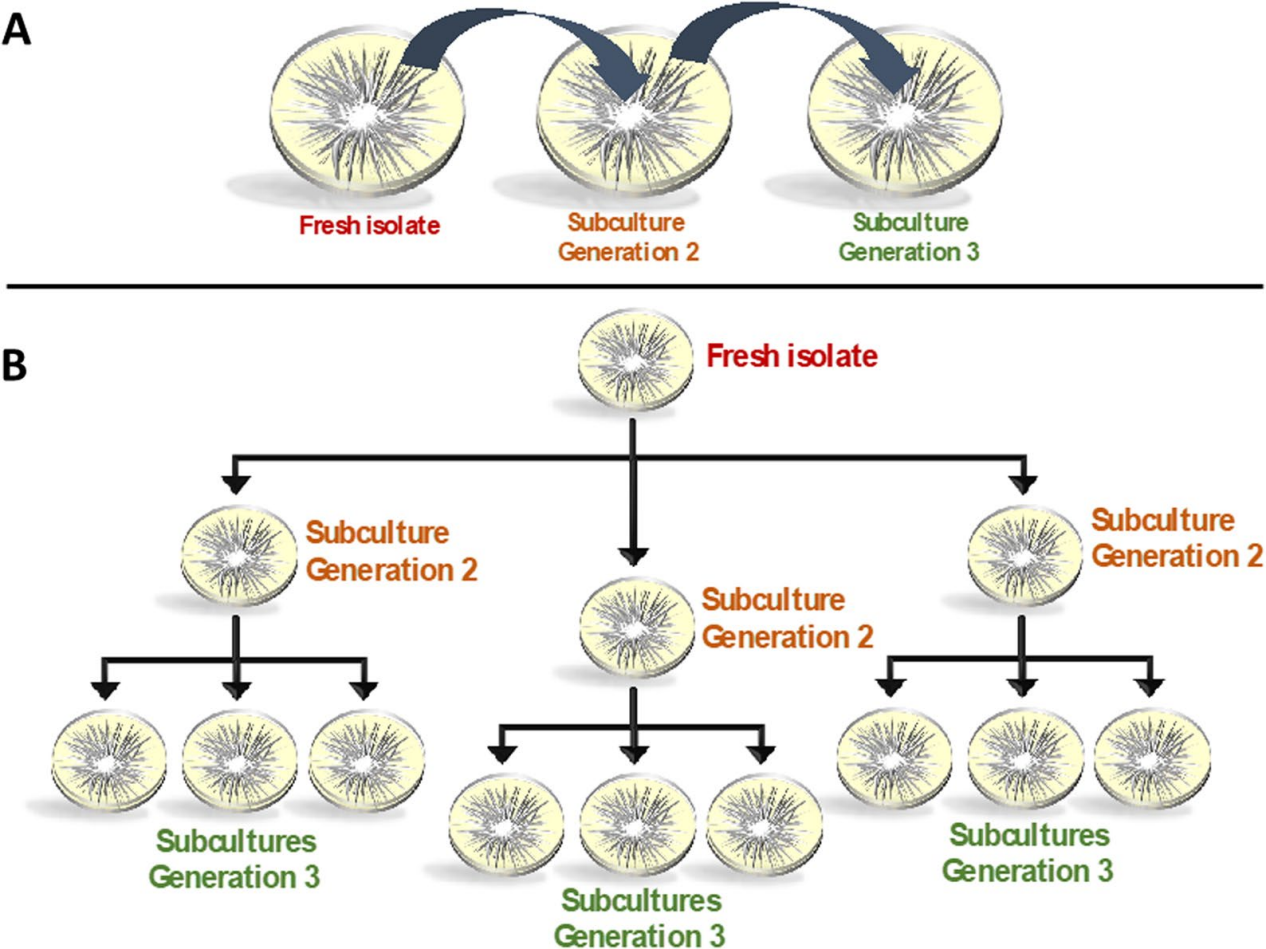
Overview of the experimental design to evaluate the consistency in bacterial presence on FON2 hyphae. **A** Hyphae from fresh FON2 isolate were transferred from edge of the growing colony to center of another plate for a total of three generations. **B** Each subculture was replicated 3 times

Ten days after-inoculation, stem slices (> 3 mm thick) from the inoculated (Fig. 1A) and non-inoculated plants were removed from above the soil line. Inoculated plants had symptoms, including leaf wilt and discoloration, consistent with Fusarium wilt infection (Fig. 1A). The tissue samples were rinsed with 10% bleach, surface sterilized with 70% ethanol and washed with sterile water, each step done for two minutes before aseptically transferring tissue to the center of a half-strength potato dextrose agar (½ PDA). The plates were incubated upright in an incubator at 27**°**C for seven days. These plates were labeled “*Generation 1*” cultures. On day seven, when hyphae had grown from diseased tissue, sterile toothpicks were used to carefully collect the hyphal tips at the edge of the colony from the surface layer of the agar media at 4 quadrants to represent 4 replicates per plate. Hyphal tip microbiota collected from sterilized toothpicks were suspended in molecular-grade water (1 mL). Then, 20μl of the FON2 water suspension was dispensed to the middle of ½ PDA plate labeled as *Generation 2*. Finally, 500μl of the water and FON2 solution was mixed with 500 μl of 60% glycerol and stored at -80**°**C for preservation, and the remaining amount was stored as DNA material for amplicon-sequencing. Of the four *Generation 2* plates, 3 plates were resampled at 4 quadrants as earlier and labeled as *Generation 3* plates. Several symptomatic plants inoculated with the isolates were initially plated with the protocol described. The first generation would produce one plate, the second generation would produce 4 plates, and the third replicate would produce 16 plates. Due to the volume of plates, a subsample of 13 suspensions from one plant was selected as shown in Fig. 2. The resulted 13 microbiota suspensions were obtained and analyzed to determine the microbial community composition.

### Microscopy

Throughout the incubation of the FON2 hyphosphere, culture plates of the three fusaria were regularly inspected for hyphal growth. Hyphal tips were examined under an upright Olympus BX60 compound microscope using 40 × /0.65 Ph2 objective. Images were acquired using an Olympus camera model LC30 (Olympus Soft Imaging Solutions GMBH, Munster, Germany). Sample images of all hyphosphere of FON2 isolates were grown on 0.5X PDA for a minimum of three days then viewed hyphal tip for bacterial interactions were viewed microscopically.

### DNA extraction and SSU rRNA library preparation

Aliquots of 200 μl of 13 suspensions were used as DNA samples which were placed at -18°C overnight and brought to room temperature three times for a freeze–thaw lysis DNA was initially quantified in a SpectraMax® QuickDrop™ Micro-volume spectrophotometer (Molecular Devices, USA) and then in a Qubit® 2.0 Fluorometer (Invitrogen, Life technologies, USA) with AccuGreen™ High Sensitivity dsDNA Quantitation Kit (Biotium, USA). The presence of fungal and bacteria DNA within the samples were assessed by PCR using established universal primers: ITS1 (5’-TCC GTA GGT GAA CCT TGC GG-3’) and ITS4 (5’-TCC TCC GCT TAT TGA TAT GC-3’) for fungal DNA, and 16S rRNA gene amplification using 27F (5’-AGA GTT TGA TCM TGG CTC AG-3’) and 1492R (5’-GGT TAC CTT GTT ACG ACT T-3’) primers for bacteria [33, 34].The identities of FON2 in these samples were confirmed by Sanger's sequencing of the ITS products by using an ABI 3730xL DNA analyzer (Applied Biosystems) by Eton Bioscience (Triangle Park, NC, U.S.A.). A nested amplification approach was used to prepare amplicon sequencing libraries for 16S rRNA. The first step of nested PCR utilized primers 27F and 1492R, samples with 16S rRNA gene amplification and were visually confirmed after electrophoresis on 1% agarose gel containing GelGreen (Biotium, USA). The second PCR reaction consisted of 16S rRNA gene primers without overhang 341F (5’-CCT ACG GGN GGC WGC AG-3’) and 785R (5’-GAC TAC HVG GGT ATC TAA TCC-3’) [35]. PCR conditions followed the KAPA HIFI Hotstart Readymix PCR kit protocol (KAPA Biosystems). Final nested PCR products were sent to Novogene (Novogene Corporation Inc., Sacramento, CA, USA) for sample preparation and 16S rRNA gene sequencing with Illumina NovaSeq PE250 platform (Illumina, San Diego, CA).

### Sequence analysis

Metabarcoding sequence analysis were processed in Mothur v.1.48.0 [36] following the MiSeq SOP pipeline with modifications [37]. The modifications introduced were based on our initial assessment with sequences obtained from the 13 hyphospheres of one of the FON2 strains, COM. Based on the initial rarefaction curves (Additional file 1: Figure S1) we determined that sequence depth of 50,000 reads per sample would suffice for hyphosphere metabarcoding. Therefore, we began the Mothur SOP pipeline with subset of 50,000 sequences with sub.sample() command. Additionally, in the initial test the align.seqs step resulted in loss of several reads which was rectified when the “flip” option was set to “flip = T” (to reverse complement the reads that did not align in the forward direction). Based on these observations, a modified pipeline incorporating these two changes was applied to the final analyses of the 39 samples (13 samples per FON2 hyphosphere). The OTUs thus obtained were classified with SILVA v132 bacterial reference trained with our v3-v4 primers.

### Statistical analysis

Mothur OTU results were analyzed and visualized using R version 4.2.1 (RStudio, Boston, MA, USA). Alpha-diversity indices were calculated within Mothur to obtain the richness and evenness measures within our samples, including Shannon, Chao, and Simpson evenness. Tabulated alpha-diversity output from Mothur was compared among isolates with ANOVA and Tukey HSD test and visualized using ggplot2, stats, and multicompview packages [38, 39]. Relative abundance stats obtained from Mothur were utilized in co-occurrence network analysis. Three different networks were generated for each of the individual hyphosphere populations. The networks were generated as Spearman correlations (coefficients > 0.7) within the Microeco package and visualized with Gephi 0.10.1 [40, 41]. FASTA output from “get.oturep” command to get list of OTU and representative sequences were utilized for Maximum-likelihood phylogenetic trees and calculated within molecular evolutionary genetics analysis program (MEGA11) were calculated with default bootstrap value of 500 and utilized within the interactive tree of life program for tree visualization of the core microbiome among isolates (iTOL v6) [42, 43]. Weighted and unweighted UniFrac were visualized with R packages phyloseq [44], tidyverse [45] and ggplot packages with maximum-likelihood phylogenetic trees made from MEGA11 program. Linear Discriminant Analysis (LDA) with logarithmic score significant value of 0.05 and LDA cut off of 3 was performed at OTU level to identify taxa that are significantly unique to our isolates hyphosphere; results were visualized with microbiomeMarker package [46].

Core microbiota associated with the hyphal tip and proximal hypha was determined by OTUs that occurred in at least 3 samples of the 39 iterations across all three isolate samples. Once the core microbiome was identified, the selected OTUs were arcsine transformed and were presented as a ternary plot to visualize the distribution of the microbial communities among our three isolates through the ggplot2 extension ggtern package [47]. Heatmaps were generated from the core microbiota to visualize the relative abundances of the OTUs among the test fungi using the phyloseq package [44]. An OTU was deemed to be core if it was observed in three or more samples within the 13 samples. These were visualized as Pearson correlation plots to distinguish positive and negative interactions within each FON2 isolates core microbiota; this was generated with the corrplot package [48]. Since the hyphosphere samples consisted of very low biomass, the DNA yield was too low to perform shotgun metagenomics analyses. Hence, the functional prediction of bacterial communities within individual hyphospheres was performed using PICRUSt2 based on the Kyoto Encyclopedia of Genes and Genomes (KEGG) database [49, 50]. The *t*-test was utilized to determine significant differences in functional potential of the hyphosphere microbiota among the three test fungal isolates with RStatix R package [51].

## Results

### Microscopy

**Fig. 3:**
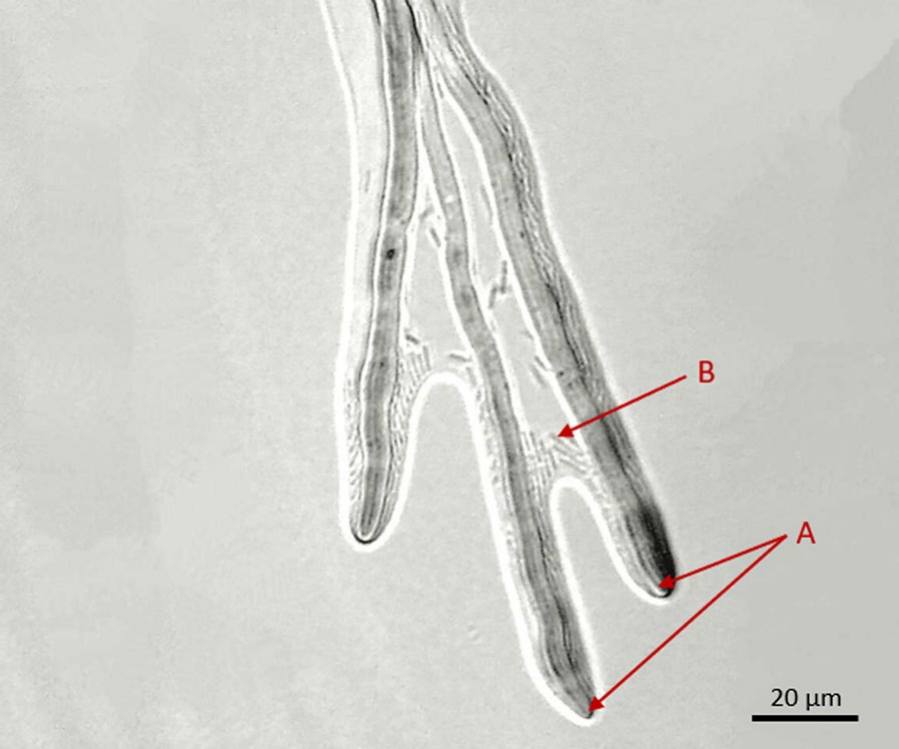
Light-microscopic images of Bacteria (B) surrounding the FON2 hyphae (A). The bacteria are freely motile and moving in the direction of hyphal growth

The microscopic image shown in Fig. 3 depicts bacteria that congregate at the elongating hyphae. Out of the four cultured plates, one plate was dedicated to observing "hyphal riding." This interplay between bacteria and hyphae was consistently observed across all isolates and subsequent generations.

### Sequencing and composition results of hyphosphere communities of FON2

NovaSeq PE250 sequencing of the 16S rRNA fragment from the V3-V4 region yielded 6,272,775 raw reads from the 39 samples. After sub-sampling, 50,000 reads per sample and processing through the modified Mothur pipeline resulted in 13,619 reads per sample classified as 1201 OTUs. The hyphosphere bacteria were classified as 5 phyla and 21 families, with *Proteobacteria* being the dominant phylum (97%) followed by unclassified bacteria (1.16%). At the family level, *Oxalobacteraceae* (39.57%), *Burkholderiaceae* (27.72%), and *Comamonadaceae* (24.47%) were the majority of OTUs identified from all samples.

**Fig. 4:**
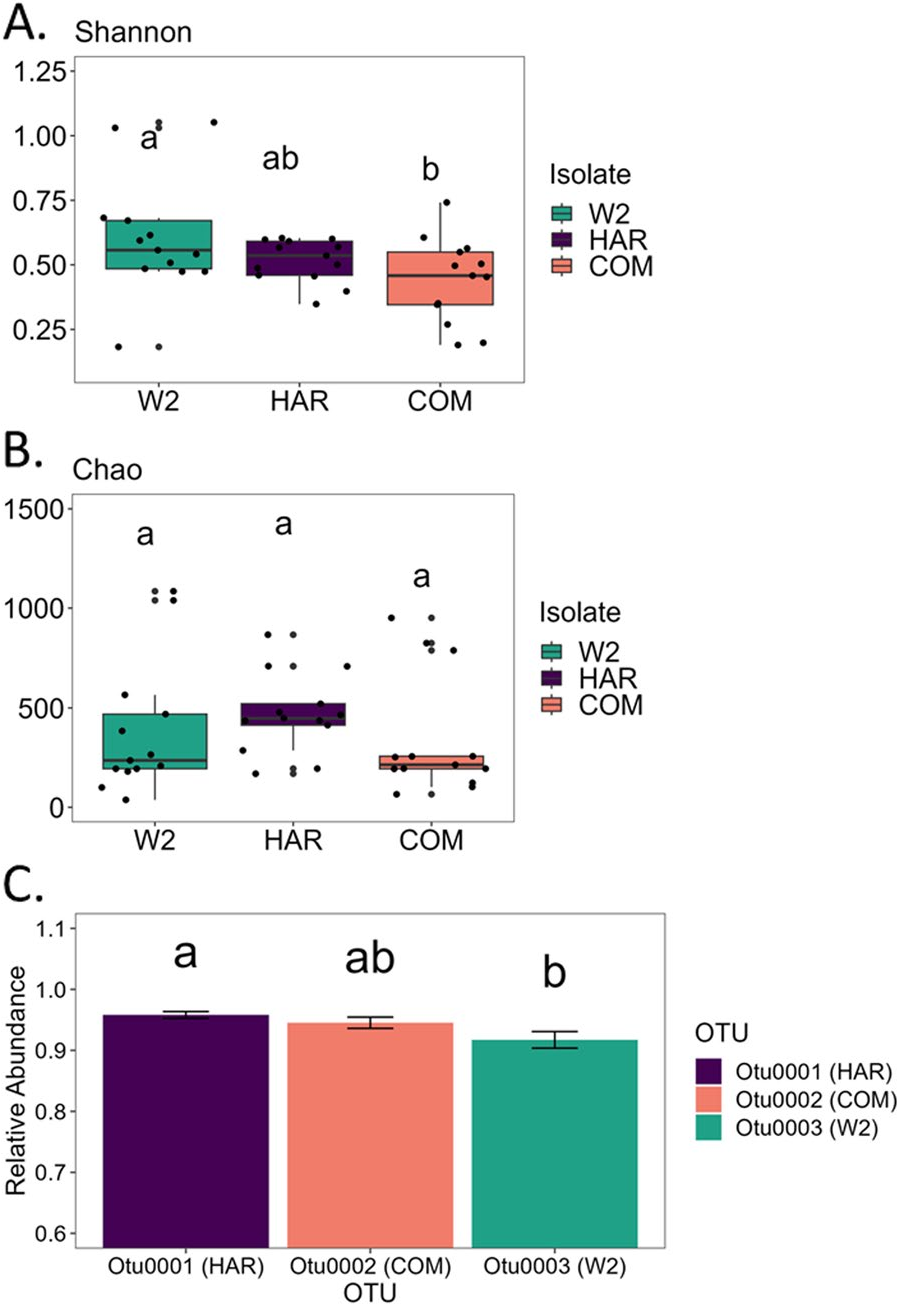
Shannon alpha diversity analysis (**A**) shows significant differences among the three isolates’ hyphosphere, while no significant differences were observed in Chao analysis (**B**). The lack of evenness that alters the Shannon index is likely due to the relative abundances of the most dominant OTU of the three hyphospheres; which were Otu0001 for isolate HAR, Otu0002 for isolate COM, and Otu0003 for isolate W2. These three dominant OTUs were significantly different by relative abundances as shown in panel C

**Fig. 5:**
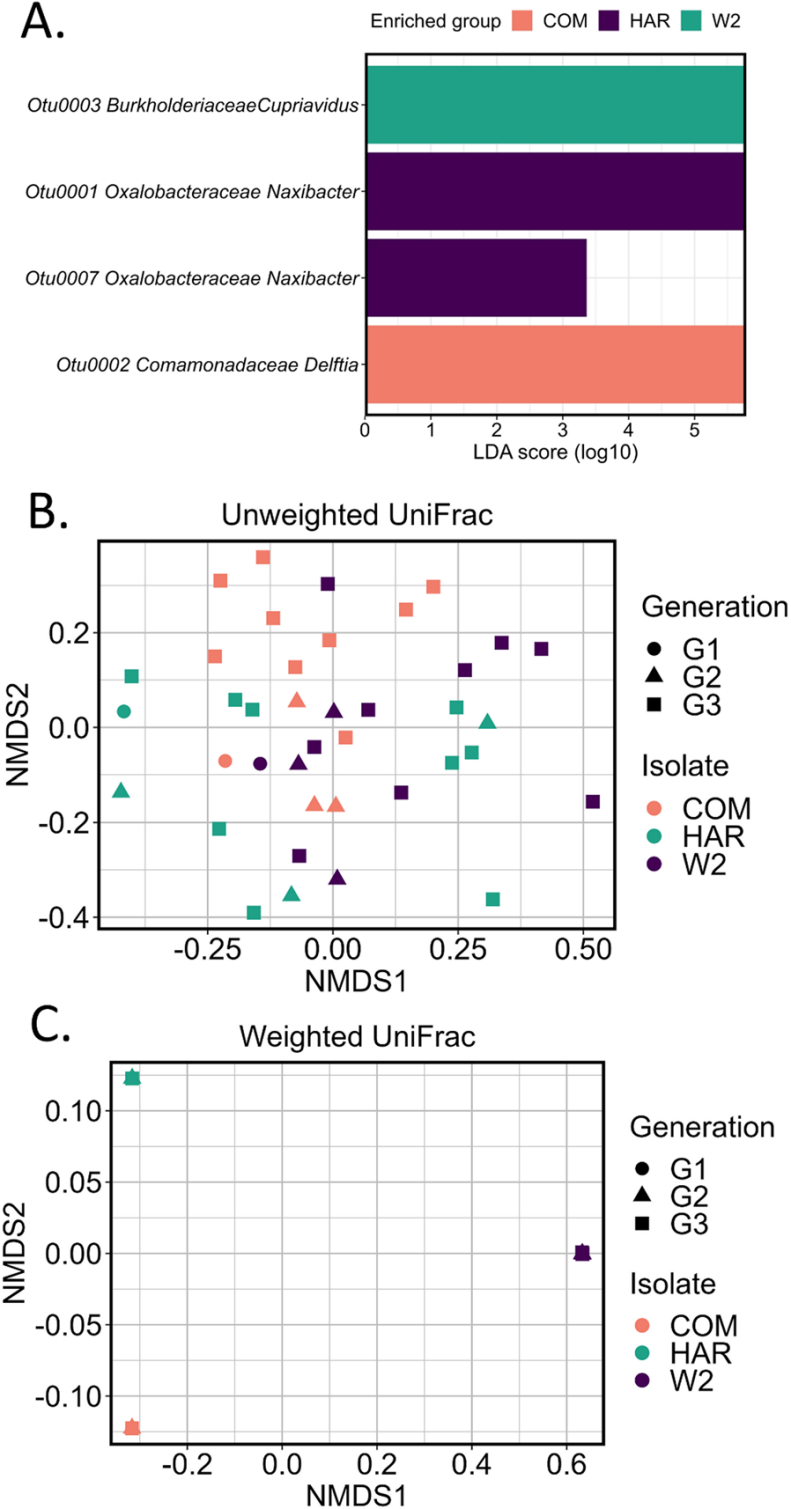
Results from LefSe (**A**) showed significant associations with four dominant OTU to each of the three FON2 isolate. While the unweighted UniFrac (**B**) does not show difference in beta-diversity among the communities, weighted UniFrac (**C**) shows strain-to-strain variations within our isolates. Neither of the analyses showed grouping based on generations. The weighted UniFrac (**C**) analysis visualized samples were similar to their own isolate. As weighted UniFrac is affected by overabundant OTUs, clustering may be due to overabundance of a single OTU of each isolate hyphosphere sample

Alpha diversity indices Shannon (Fig. 4A), Chao (Fig. 4B), Simpson evenness and observed (Additional file 1: Figure S2A, B) were utilized to compare the overall diversity among the three FON2 isolates hyphosphere. The Shannon diversity analysis indicated significant differences among the diversity of the FON2 hyphosphere. When comparing COM and W2, W2 had significantly higher Shannon values (*P* < 0.05) whereas COM compared to HAR and HAR to W2 were not significantly different. This trend wasn’t consistent with the Chao richness estimate results, which showed no significant differences among the FON2 isolates. To further evaluate this inconsistency, analysis of variance (ANOVA) and the Tukey HSD (honestly significant difference) test were utilized with the dominant OTU of each FON2 isolate (Fig. 4C). The relative abundance of the Otu001 (dominant OTU of HAR FON2 isolate) was significantly higher than Otu003 (dominant OTU of W2) (*p*^adj^ = 0.017), whereas Otu002 (dominant OTU of COM) was not significantly different from either one. LDA was conducted with LefSe at the OTU level to identify unique individual bacteria to FON2 isolate (Fig. 5A). We hypothesized a reduction in alpha-diversity with each generation until the fungal selection of bacterial community is stabilized. Hence, we found no significant difference in Shannon diversity, Simpson evenness, and Chao species richness analysis when comparing the alpha-diversity of the three generations (Additional file 1: Figure S3A–C).

Histograms of the LDA analysis showed that Otu002 (*Comamonadaceae*) was highly correlated with COM, while W2 was significantly correlated with Otu003 (*Burkholderiaceae*). Furthermore, Otu0007 (*Oxalobacteraceae*), and Otu001 (*Oxalobacteraceae*) were significantly correlated with HAR. With the exception of Otu007, which was significantly associated with HAR, the remaining 3 significant OTUs had the largest by relative abundance for their respective hyphosphere communities (Fig. 5A). Additionally, all four OTUs that showed significance in LDA (and hence all the dominant OTUs) belonged to the order *Burkholderiales*. Unweighted UniFrac analysis of the hyphosphere communities could not be clustered based on FON2 hosts nor by generations. Therefore, the community structure from the FON2 isolates is not dissimilar even though the fungal host background has temporal and spatial differences (Fig. 5B). While weighted UniFrac points were clustered closely based on FON2, Isolate clustering may be affected by an overabundant OTU unique to each isolate as shown in Fig. 5C. Therefore, from the two UniFrac analyses, unweighted denotes community membership is similar while weighted visualizes the effects of population abundance dissimilarity.

### Core hyphosphere microbiota shared among FON2 Isolates

**Fig. 6:**
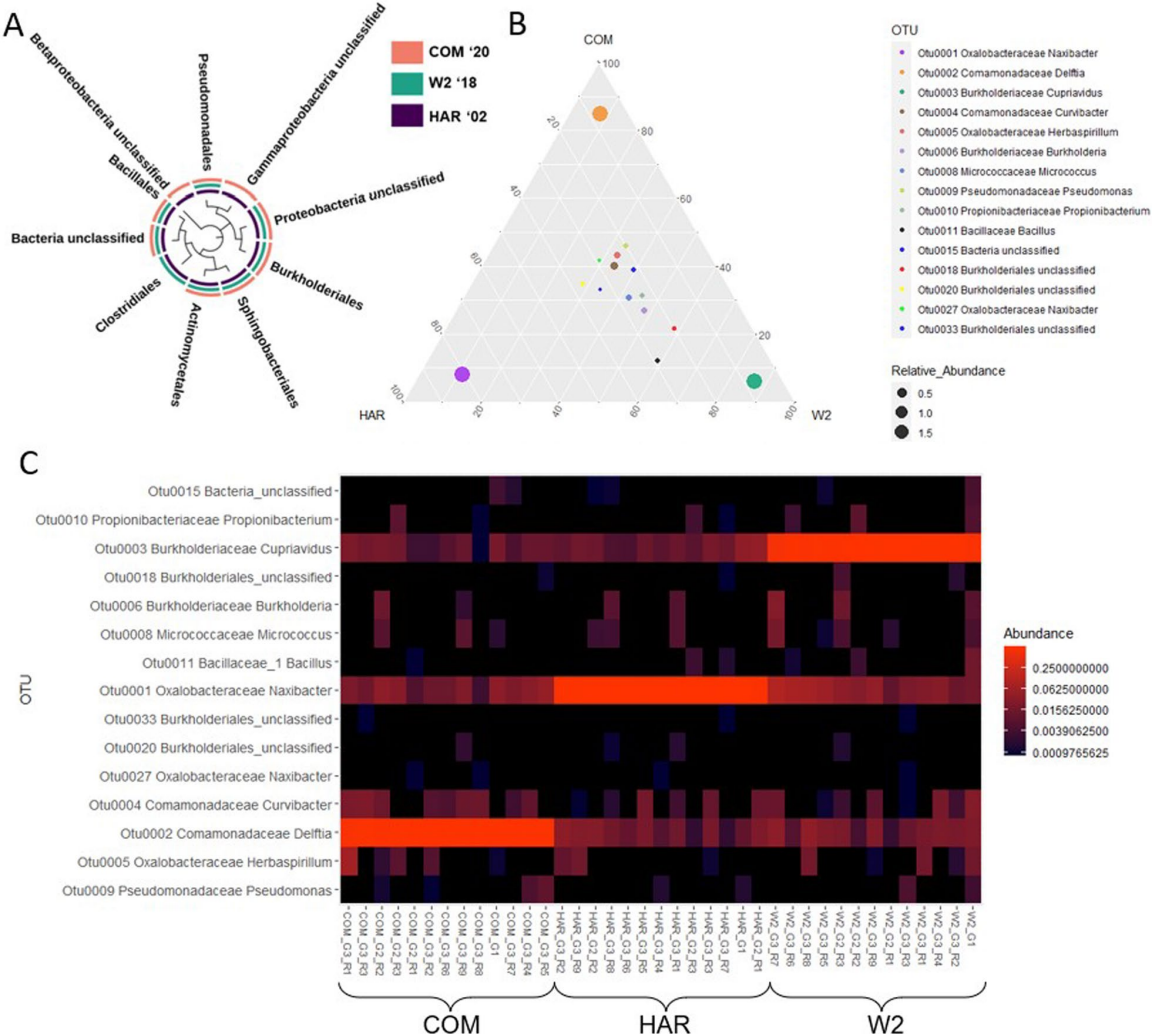
Phylogenetic relationships of orders with colors on the arc denoting the presence of orders within FON2 isolates samples. At the order level *Pseudomonadles*, unclassified proteobacteria, unclassified bacteria, *Actinomycetales*, *Sphingobacteriales*, and *Burkholderiales* were shared among all FON2 Isolates (**A**). At the OTU level ternary plot (**B**) visualizes a core bacterial strain associated equally among the three isolates in the center whereas the corners have a single dominant OTU with higher abundance showing a stronger correlation to their particular isolate. The heatmap (**C**) visualizes relative abundances of the core OTUs across all three generations of the three hyphospheres. A single OTU consistently dominates each hyphosphere (i.e. OTU002 with COM and OTU001 with HAR and OTU003 with W2)

Of the ten orders that were identified, *Pseudomonadles*, unclassified proteobacteria, unclassified bacteria, *Actinomycetales*, *Sphingobacteriales*, and *Burkholderiales* were found within all of the three FON2 strain hyphospheres (Fig. 6A). The core microbiota of the FON2 hyphosphere resulted in a total of 15 OTUs, which made up 96% of the total number of OTUs. A ternary plot was used to visualize the distribution of the 15 OTUs among the three FON2 hosts (Fig. 6B). The ternary plot demonstrated that Otu004 (*Comamonadaceae*), Otu005 (Oxalobacteraceae), Otu006 (*Burkholderiaceae*), Otu008 (*Micrococcaceae*), Otu009 (*Pseudomonadaceae*), Otu010 (*Propionibacteriaceae*), Otu0015 (Bacteria unclassified), Otu0020 (*Burkholderiales*), Otu0027 (*Oxalobacteraceae*), and Otu033 (*Burkholderiales*) were clustered towards the center, suggesting they might be central to FON2 hyphosphere functions, regardless of spatial and temporal differences. Otu0018 (*Burkholderiales*) and Otu0011 (*Bacillaceae*) were relatively higher in W2 host compared to COM and HAR. As mentioned earlier, outliers Otu001, Otu002 and Otu003 were the most abundant OTUs and found within all the samples while they have a strong relationship with one host.

This strong relationship of a single OTU dominance with the FON2 host is seen within the heatmap (Fig. 6C) and is shown as a high relative abundance value, further validating results observed from the ternary plot. The heatmap displays more details of Otu001 (*Oxalobacteraceae*), Otu002 (*Comamonadaceae*), and Otu003 (*Burkholderiaceae*) presence within all samples and those of the other core OTUs. This again agrees with the LDA results, as Otu001 has a significant relationship with HAR, Otu002 with COM, and Otu003 with W2. Further LDA results of Otu007 indicate a significant relationship with HAR (Fig. 5).

### Co-occurrence and correlation analysis of FON2 isolates hyphosphere

**Fig. 7:**
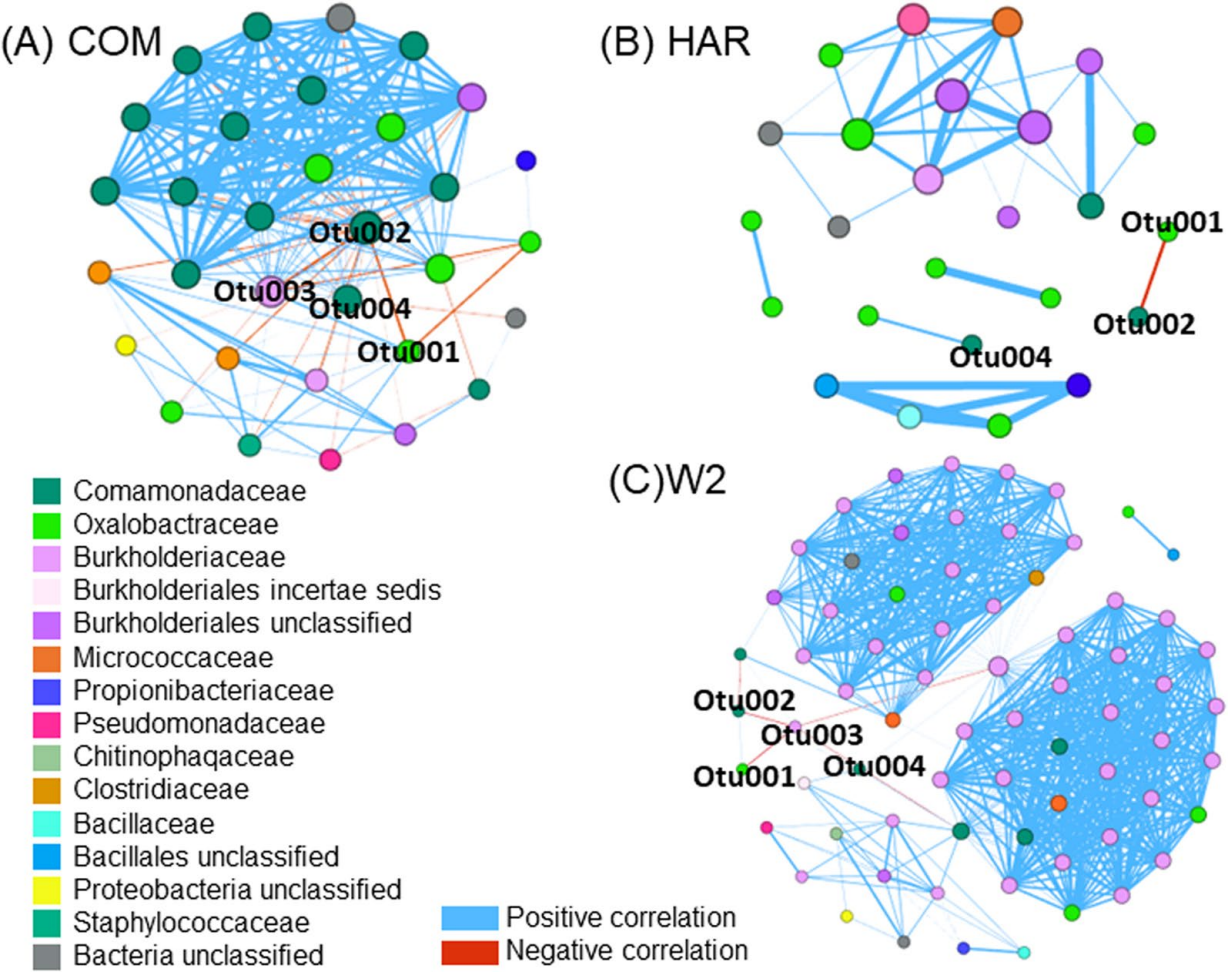
Spearman correlation analysis of COM (**A**), HAR (**B**) and W2 (**C**) hyphospheres found the major OTUs (Otu001, Otu002, Otu003) to have a negative correlation to the majority of OTUs among all three co-occurrence networks. Furthermore, the three major OTUs are always proximally together and away from the major clusters. These results may be due to an overabundance of OTUs as well as having a separate function within the community

**Fig. 8:**
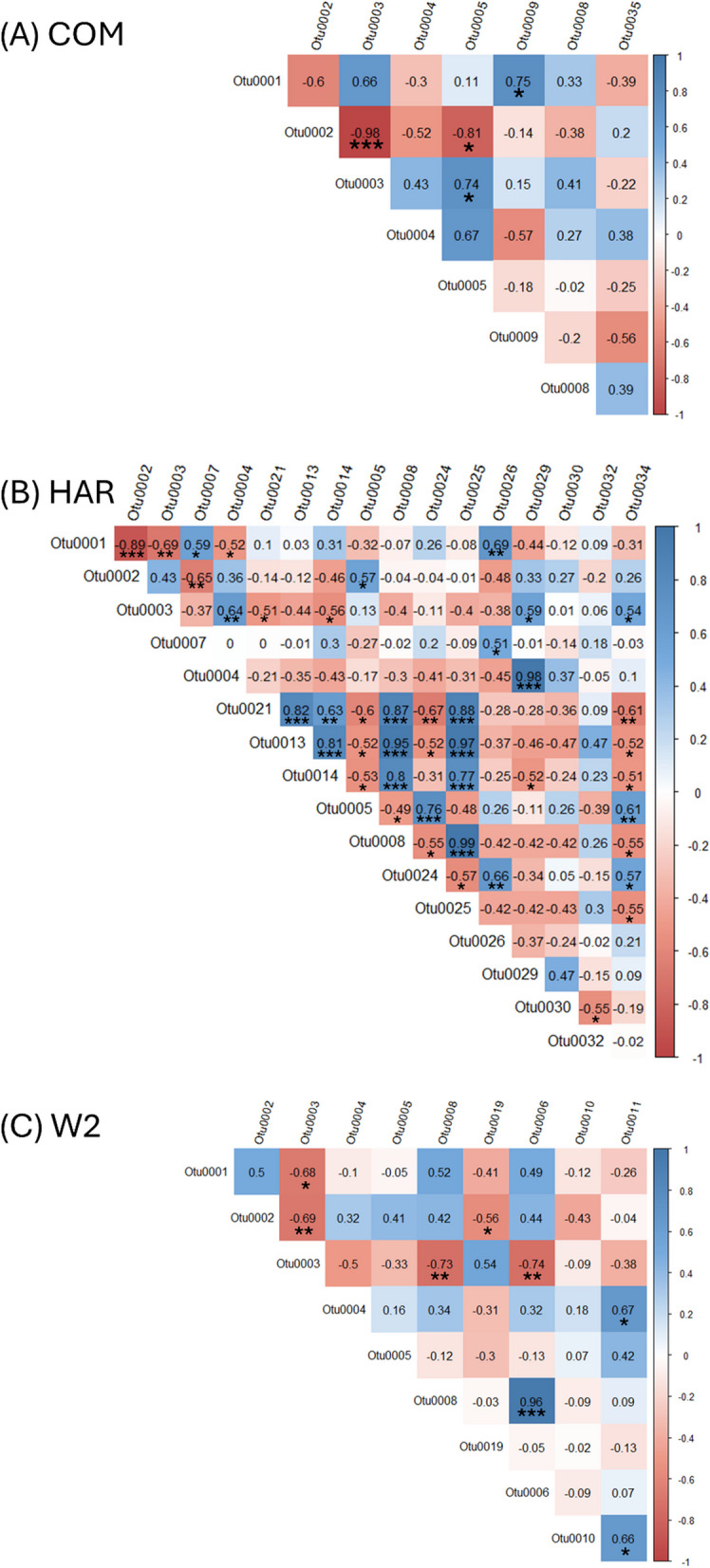
Pearson correlation plots (**A**–**C**) visualizing the core microbiome interaction within each individual isolates hyphosphere (significant values *p < 0.05, **p < 0.01 and ***p < 0.001). There was a negative correlation found with the dominant OTU (COM with Otu0002, HAR with Otu0001 and W2 with Otu0003) to the rest of the microbiota

Individual FON2 isolate's microbiomes were analyzed through co-occurrence networks to compare possible host-specific assemblies (Fig. 7). Within the COM hyphosphere network, there were 215 edges (33 negative and 182 positive edges) and 32 nodes, while HAR had 1 negative and 40 positives edges and W2 had a total of 732 edges (4 negative and 728 positives). Core OTU of individual isolates were identified to analyze host-specific assemblies, identifying 8 OTUs within COM, 17 within HAR, and 10 with W2. The correlations among the individual FON2 core microbiota were compared, as shown in Fig. 8. Similar to results from the co-occurrence model with Spearman correlation, there was a negative interaction among Otu001, Otu002, and Otu003. The COM dominant Otu002 was negatively correlated with the other 6 Otus, the W2 dominant Otu003 was negatively correlated with 7 OTUs and positively correlated with one (Otu0019), and HAR dominant Otu001 was negatively correlation with 8 other OTUs and positively correlated with 4 OTUs (Otu0014, Otu007, Otu0026, and Otu0024). Within HAR there was a positive cluster of Otu0025, Otu008, Otu0021, and Otu0013 that was negatively correlated with the dominant Otu001; therefore, may be a different assembly within the hyphosphere. Although there was a robust negative correlation among the dominant OTUs from the correlation plot, most of the co-occurrence network was positively correlated, displaying a distinct community structure.

#### Predictive functions of the hyphosphere

**Fig. 9:**
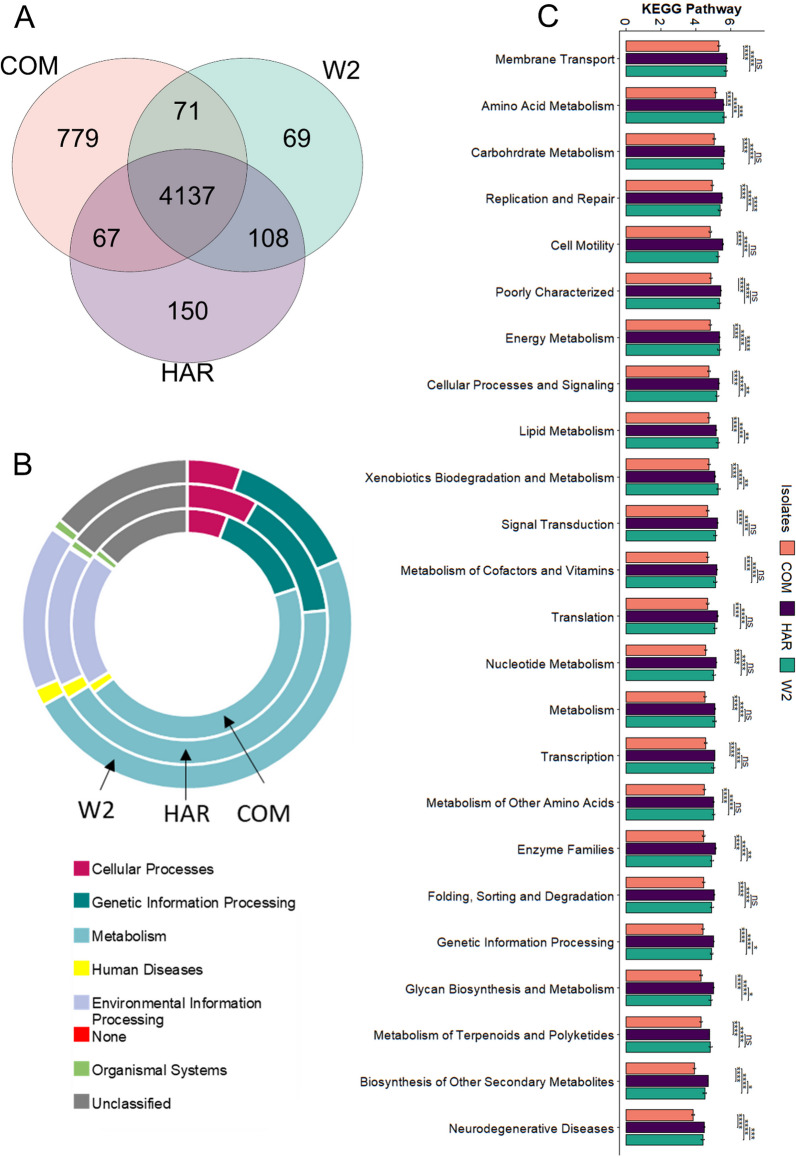
The PICRUSt results predicted different functions from the KEGG database. At level 3 (**A**) FON2 COM had the most unique predicted pathways, followed by HAR and, the least from W2 (**A**). A donut plot was used to visualize level 1 (**B**) KEGG results from the three isolates hyphosphere. At level 2 (**C**), the top 24 PICRUSt results found COM had significantly lower than W2 and COM. Whereas HAR was significantly higher in 16 of the 24 functions predicted and W2 with 8 significantly higher functions than COM and HAR

PICRUSt analysis from 16S rRNA sequencing referenced to the KEGG database resulted 5,380 predicted KEGG Orthology (KO) groups. Of these KOs, 779 were unique to COM, 150 to HAR, 69 unique to W2 and 4,137 were shared among all three as seen in Fig. 9A. The predicted categories of major functions within the hyphosphere, as shown in Fig. 9B, included metabolism (COM at 44.99%, W2 at 48.22% and HAR at 42.71%), environmental information processing (COM at 18.77%, W2 at 16.24% and HAR at 16.37%) and unclassified (COM at 13.73%, W2 at 14.04% and HAR at 14.70%). In level 2 of predicted function, we chose to visualize the top 24 to compare among the hyphospheres of the three isolates. Within the COM hyphosphere, all 24 were significantly lower than that of HAR and W2. FON2 isolate HAR’s hyphosphere had 8 predicted functions that were significantly higher compared with W2. These predicted functions were replication and repair, energy metabolism, cellular process and signaling, enzyme families, genetic information processing, glycan biosynthesis and metabolism, biosynthesis of other secondary metabolites and neurogenerative diseases. Within W2, four functional genes were significantly higher than those found in HAR (amino acid metabolism, lipid metabolism, xenobiotics biodegradation and metabolism and metabolism of terpenoids and polyketides. The overall results indicated the hyphosphere of COM (the most recent isolate) is predicted to have less shared activity compared with HAR (the oldest isolate) and W2 but has more unique unshared functions. Within the predicted results associated with terpenoids and polyketides and ubiquinone and other terpenoid-quinone biosynthesis functions, 137 reference enzyme commission pathways (EC) were identified. The predicted EC included ubiquinone and other terpenoid-quinone biosynthesis, sesquiterpenoid and triterpenoid biosynthesis, streptomycin biosynthesis, carotenoid biosynthesis, lysine degradation, caprolactam degradation and zeatin biosynthesis.

## Discussion

Pathobionts associated with the plant pathogens represent a major component of factors leading to disease [17, 51]. Hence, the pathogen and their pathobionts form the microbial constituents of a pathobiome. However, their precise contribution to pathogenicity remains uncertain [52]. The hyphosphere is a micro-niche that a soil-borne pathogen manipulates to enable its pathogenicity through the production of extracellular enzymes and secondary metabolites [53–55]. Thus, the bacterial microbiota present in this zone and attached to the fungal hyphae are an essential component of the pathogen’s lifecycle. These bacteria may or may not contribute directly to the pathogen’s virulence, either to encourage or alleviate the disease. In this study, we identified a core hyphosphere microbiota characterizing the pathobiome of FON2. This marks the first step towards unraveling the microbiome functions that includes the pathogen’s microbial community assemblage.

We suggest that if the hyphosphere bacterial composition of FON2 remains consistent across successive subcultures on media and exhibits similarity among various strains, it is highly likely that these bacteria play a functional role either to promote virulence, or even potentially as a parasite or commensal within the assemblage. As these bacteria are part of a pathogen’s assemblage, as per the pathobiome concept [17, 56], this should represent a pathobiome. Therefore, we tested our hypothesis, trying to mimic natural infection, using three unique isolates with different spatial and temporal backgrounds. By placing the inoculum of these isolates near the rhizosphere soil of watermelon seedlings without disturbing plants, as compared with root dipping inoculation. Therefore, there was less root disturbance and a more natural situation which allowed the pathogen to migrate towards the host root. Once disease symptoms were visible, stem tissue was cultured for FON2 and its candidate pathobiome. It should be noted that in common plant pathology protocols, the stem tissues are placed on media containing antibiotics (generally streptomycin) to prevent bacterial growth. However, we avoided the use of antibiotics to ensure that the co-occurring bacteria riding the pathogen’s hypha was not inhibited in transfers. As plant residue acts as pathogen inoculum in subsequent years [57]. Hence, we hypothesize that the pathogen’s hyphosphere bacteria from the diseased tissues will represent the pathobiome relevant to the disease.

Further results from 16S rRNA sequencing identified prominent OTUs that may have an underlying function within the hyphosphere microbiome. Simpson evenness (S3) and Shannon index (Fig. 3A) were significantly different among the different isolates whereas Chao diversity was relatively unaltered. We suggest that the difference in evenness is driven by the most abundant OTUs of each of the three hyphosphere, which make up more than 80% of the relative abundances. To test this, the abundance of the major OTU from each isolate were tested via ANOVA and Tukey HSD analysis revealing their abundance is significantly different from each isolate. This overabundance of a particular OTU was seen to be associated with other aspects of our results, such as the co-occurrence network analysis and the correlation plots. The co-occurrence analysis visualized a negative correlation with the dominant OTUs from each isolate. This same interaction was reconfirmed in the correlation plot as the majority of bacteria had a negative correlation to the three major OTUs.

These particular highly abundant OTUs are significantly associated with a single FON2 i.e. COM with Otu002, W2 with Otu0003 and HAR with Otu0001, as also seen in our LefSe analyses (Fig. 4A). Similar results are from the ternary map visualizing a similar abundance of central core hyphosphere microbiota associated with the three isolates except for one dominant OTU strongly associated with a particular isolate. This pattern was demonstrated once more in the heatmap, as the most abundant OTUs were present within a particular FON2 isolates hyphosphere and was consistent throughout the isolate’s samples regardless of repetition from re-culturing and differences in host fungi’s genetic backgrounds [32].

The PiCRUSt tool predicted several potential pathways related to plant defense, development, and antimicrobial functions. Of the identified EC pathways associated with plant defense against plant pathogens, including pathways for ubiquinone and other terpenoid-quinone biosynthesis [58, 59], sesquiterpenoid and triterpenoid biosynthesis [60, 61] and carotenoid biosynthesis[62]. Other plant defense associated pathways identified within the hyphosphere included zeatin biosynthesis, which is noteworthy with the finding that the *Plasmodiophora brassicae* converts adenine to trans-zeatin to produce cytokinin which assist in gall formation in the host [63, 64]. However, it is unknown how this may be beneficial within the pathobiome is unknown and should be researched further. Lysine degradation was predicted within the hyphosphere microbiome. A previous study associated the addition of cadaverine affected lysine degradation and reduced biofilm formation with the opportunistic pathogen *Pseudomonas aeruginosa* and significantly increased planktonic movement [65]. Furthermore, lysine degradation has been identified in the fungal plant pathogen *Ustilago maydis* but the mechanism for pathogen success warrants further research [66, 67]. Streptomycin biosynthesis was also identified within the hyphosphere. *Fusarium oxysporum* f.sp*. cubense* revealed that endohyphal bacteria that were difficult to remove with antibiotics (ampicillin and chloramphenicol) [68]. Suggesting that the antimicrobial resistance may be occurring because of a hyphal barrier from genetic resistance or from a unknown chemical interaction.

In this study, the core microbiota was dominated by the order *Burkholderiales* (i.e. OTU001 *Oxalobacteraceae Naxibacter*, OTU002 Comamndaceae *Delftia* and OTU003 *Burkholderia Cupriavidus)*. Strains from this order have been previously shown to occur in the hyphosphere as well [69]. *Burkholderia terrae* BS001, from hyphosphere of *Lyophyllum,* was observed to move along with hyphal growth but also developed biofilms around the hyphae [7]. This strain was also found to use type III secretion system (T3SS) for hyphal adhesion [70]. The bacteria utilized this relationship to exchange nutrients and impart protection against antifungal agents [71]. It remains to be seen whether the hyphal-riding bacteria in our study can also impart fungicide resistance to Fusaria. Indirect evidence suggests that a strain of *Burkholderia terrae* BS001 that moves along the hypha of two *Fusarium oxysporum* isolates (Fo47 and Foln3) can also impart fungicide resistance to these strains of Fusaria [68].

Other hyphal-associated bacteria that were observed as part of the FON’s pathobiome was *Oxalobacteraceae,* which was previously associated with arbuscular mycorrhizae [63] and also colonizing the hyphae of *Pythium aphanidermatum,* with an association of increased virulence of *Pythium* on seeds [72, 73]. Therefore, we suggest from our results and previous research support *Oxalobacteraceae Naxibacter* sp., *Comamonadaceae Delftia* sp. and *Burkholderiaceae Cupriavidus* sp. as organisms associated with the hyphosphere, i.e. hyphal riders.

Plant pathogens and their interactions within the pathobiome is a relatively new concept [17]. Although this study has identified the shared core microbiota within the hyphosphere, research is necessary to investigate if other FOSC also have a consistent hyphal-associated microbiota. Additionally, there is a need to differentiate the pathobiome community from the plant host community under dysbiosis. Another question is whether these interactions can be replicated in situ, by isolating core bacteria and reintroducing them to the pathogen. Finally, these interactions should be profiled for their metabolites. Through metabolomic profiling the presence and absence of the hyphosphere bacterial microbiota may assist in describing their roles within the pathobiome and within the host infection process.

Since the goal of the study was to examine only the bacteria that are transmitted by the fungal hyphae, samples were only collected at the tips of an actively growing hypha. This in turn yielded very low biomass per sample from which DNA could be extracted. Such low biomass samples cannot be used in conventional DNA extraction protocols without losing the product. The freeze–thaw method overcomes this hurdle. It is not unlikely that the freeze–thaw method could have introduced bias against Gram-positive organisms [74]. However, the reads from the hyphosphere’s included Gram-positive bacteria such as Otu008 *Micrococcus,* Otu010 *Propionibacterium,* and Otu011 *Bacillus*.

Another challenge was the replication of one plant per isolate. One plant produced several replicates of hyphosphere samples that grew exponentially with every generation. This method posed a challenge in the workload when working with several isolates simultaneously. Despite the challenge in this method, the one plant per isolate result confirmed a shared core microbiota of the same 13 OTUs consistently recruited among several generations and different isolates of temporal and spatial background. Therefore, more plants may be sampled, and fewer generations may be required.

Our study establishes the core bacteriome associated with the hypha of pathogenic FON2. This pathobiome stayed consistent through subcultures. The community structure by beta-diversity did not separate the communities obtained from the three different strains. All these results point to a defined hyphosphere pathobiome of FON2. The use of hyphosphere as a proxy to pathobiome provides future researchers with a tool to specifically bait out the pathobiome assemblage. Such a tool will enable understanding the pathogen’s lifestyle and the processes in soil micro niches that lead to infection.

## Conclusion

In this study, we characterized the core microbiota associated with the hyphal tip of plant pathogen FON2. Closely related members of our core microbiota groups have been previously identified as hyphal-associated and now additional taxa of *Burkholderia, Bacillus* and *Pseudomonas* genera to be associated with hyphal interactions. As this specialized niche is the point of entry for root infection, this microbiota may contribute to essential functions of the pathobiome and may assist in disease success. Future studies that investigate the role of pathogen-associated microbiota associated with host infections are warranted. Results from our study emphasizes the importance of the pathobiome paradigm, that is, pathogens should not be characterized as lone invaders, but instead, they recruit and manipulate the hosts' established microbiota to form a pathobiome.

### Supplementary Information

**Additional file 1.** Supplementary Figures.

## Data Availability

Sequences were submitted to the SRA (bioproject PRJNA1032089).
