# Supplementary material for: Core hyphosphere microbiota of Fusarium oxysporum f. sp. niveum
Source: Environ Microbiome. 2024 Mar 9;19:14. doi: 10.1186/s40793-024-00558-5 (PMC10924372; doi:10.1186/s40793-024-00558-5)
Supplement: Supplementary file 1 — Additional file 1. Supplementary Figures. [file 40793_2024_558_MOESM1_ESM.docx]

SUPPLEMENTARY MATERIAL

**Core hyphosphere microbiota of** ***Fusarium oxysporum* f. sp. *niveum***

Vanessa E. Thomas, Sanjay Antony-Babu*

Department of Plant Pathology and Microbiology, Texas A&M University, College Station, TX 77843, USA

*Corresponding author: Sanjay Antony-Babu, E-mail: [sanjay@tamu.edu](mailto:sanjay@tamu.edu)

This file contains:
Supplementary Figures: 1 to 3


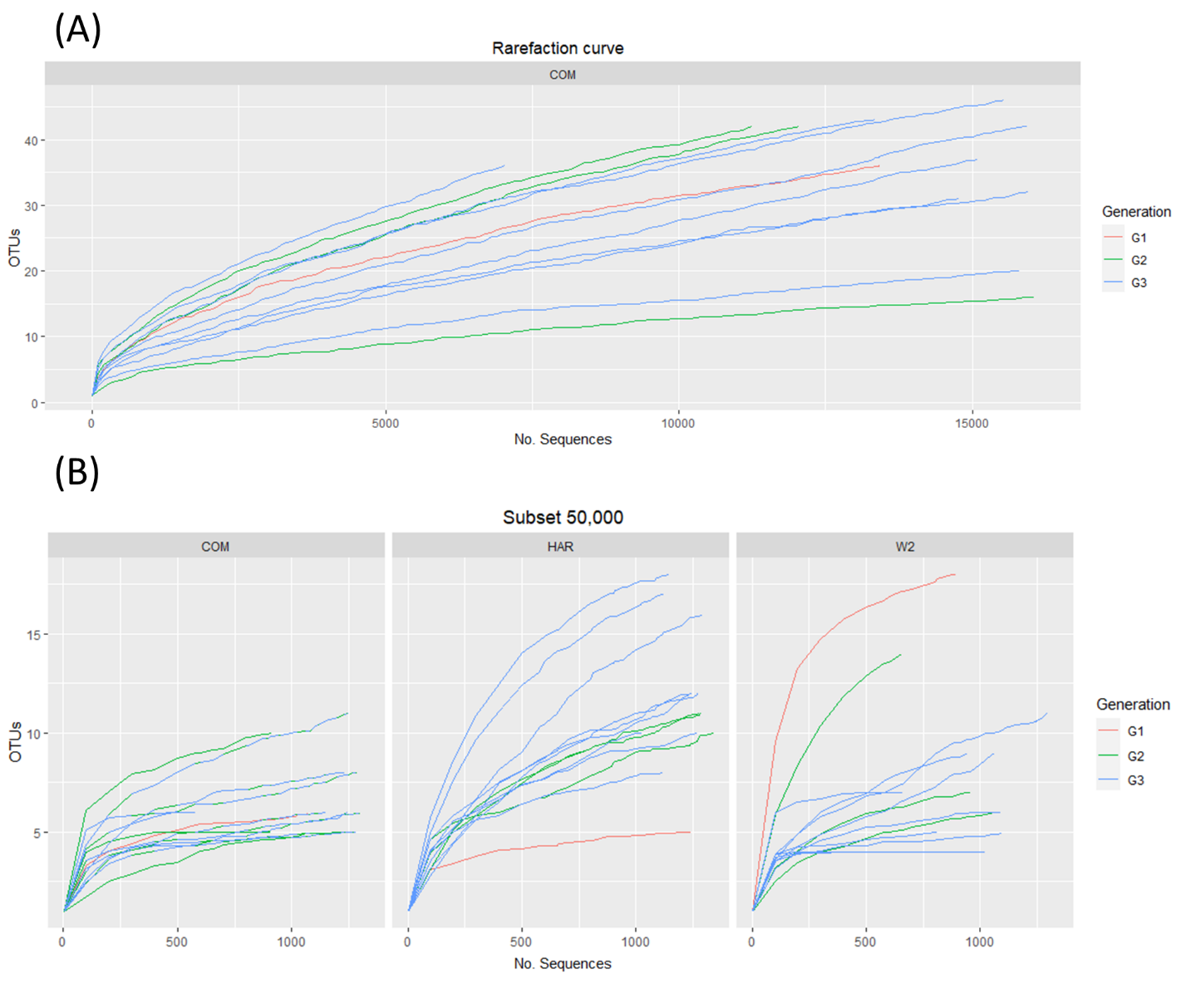


Figure S1. Within the Mothur analysis we first subset just the FON2 COM isolate samples, through rarefaction curve (A) we observed that at 50,000 sequences would provide equal coverage to all samples tested. We reanalyzed at a subset of 50,000 sequences at the beginning of the Mothur SOP pipeline, resulted sequences with all samples were visualized as a rarefaction curve (B) to confirm adequate depth to all samples.


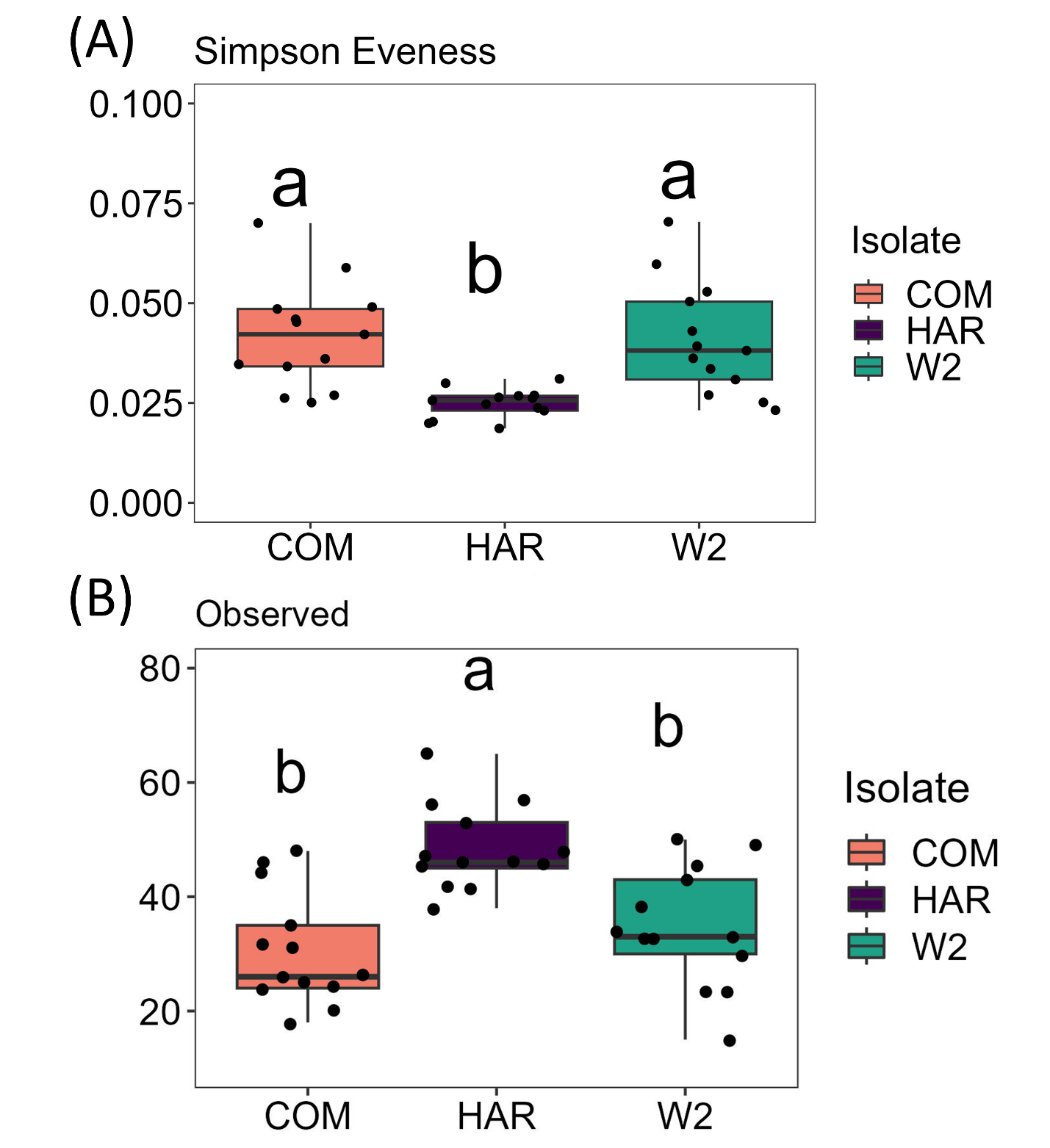


Figure S2. Alpha diversity indices from our isolates analyzed with Simpson evenness (A) found COM and W2 to have significantly similar evenness while HAR isolates did not.


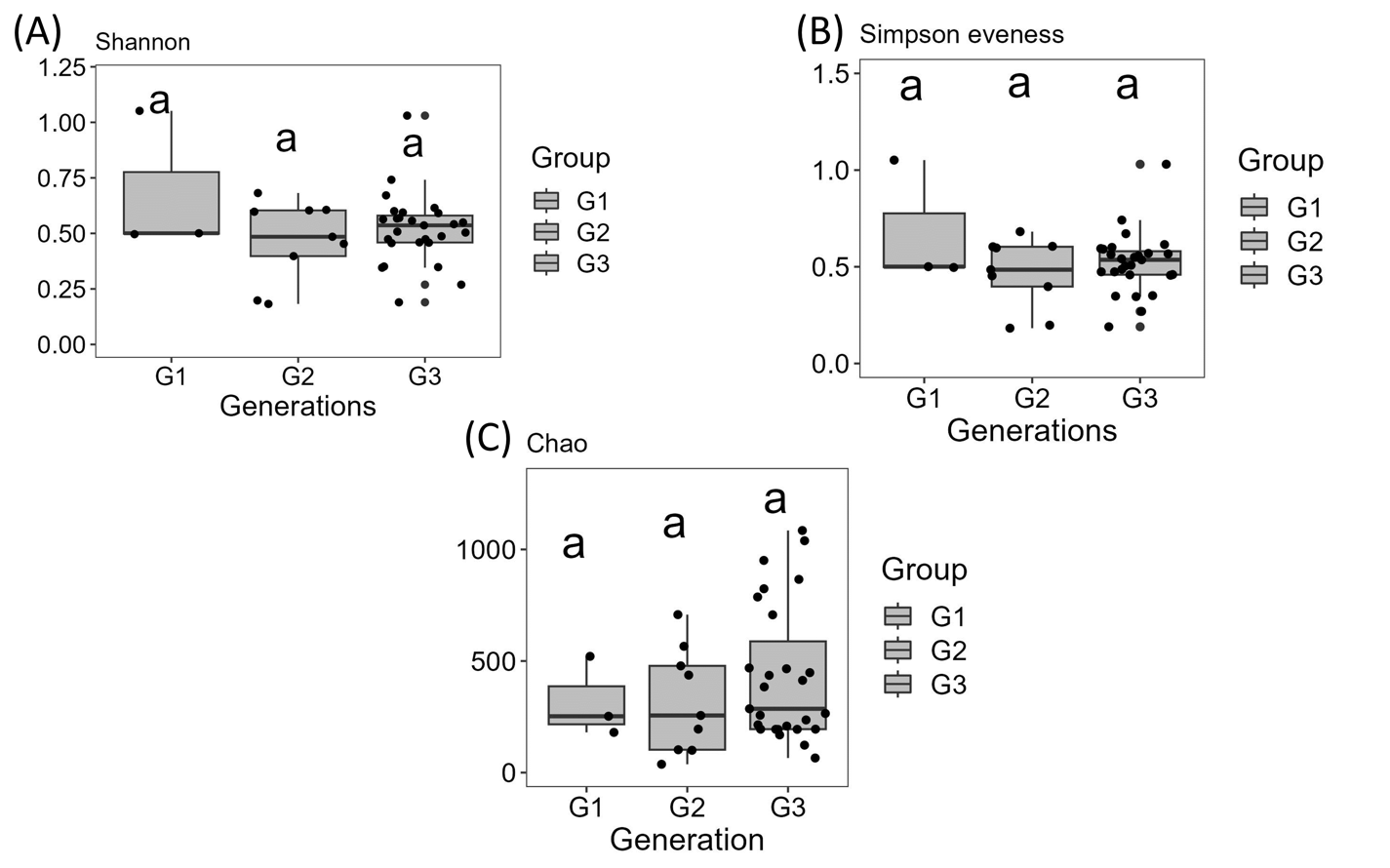


Figure S3. Alpha diversity was utilized to compare differences among the generations. Using Shannon (A), Simpson evenness (B) and Chao (C) found no differences among the diversity, abundance, and evenness of the generations of all FON2 hyphosphere communities.
